# Differential sequential patterns supporting insulin therapy of new-onset type 1 diabetes

**DOI:** 10.1186/s12938-015-0004-x

**Published:** 2015-02-21

**Authors:** Rafał Deja, Wojciech Froelich, GraŻyna Deja

**Affiliations:** Department of Computer Science, Academy of Business in Dabrowa Gornicza, Cieplaka 1c, Dabrowa Gornicza Poland; Institute of Computer Science, University of Silesia, Bedzinska 39, Sosnowiec Poland; School of Medicine in Katowice, Department of Pediatrics, Medykow 16, Katowice Poland

**Keywords:** Data mining, Medical patterns, Diabetes mellitus

## Abstract

**Background:**

In spite of numerous research efforts on supporting the therapy of diabetes mellitus, the subject still involves challenges and creates active interest among researchers. In this paper, a decision support tool is presented for setting insulin therapy in new-onset type 1 diabetes.

**Methods:**

The concept of differential sequential patterns (DSPs) is introduced with the aim of representing deviations in the patient’s blood glucose level (BGL) and the amount of insulin injections administered. The decision support tool is created using data mining algorithms for discovering sequential patterns.

**Results:**

By using the DSPs, it is possible to support the physician’s decisionmaking concerning changing the treatment (i.e., whether to increase or decrease the insulin dosage). The other contributions of the paper are an algorithm for generating DSPs and a new method for evaluating nocturnal glycaemia. The proposed qualitative evaluation of nocturnal glycaemia improves the generalization capabilities of the DSPs.

**Conclusions:**

The usefulness of the proposed approach was evident in the results of experiments in which juvenile diabetic patients actual data were used. It was confirmed that the proposed DSPs can be used to guide the therapy of numerous juvenile patients with type 1 diabetes.

## Introduction

In recent years, the number of cases of diabetes mellitus has increased significantly [1]. Therefore, controlling the concentration of glucose in the patient’s blood is still an important problem that is being investigated by many researchers. The problem is a challenge since the strict medical procedure for controlling BGL cannot be defined [2]. The main reason is the high variability between people and the numerous different initial clinical states of patients after they are admitted to the hospital. This especially concerns the onset of diabetes, when the patient’s state is unstable. For that reason, the existing medical approach to determining the appropriate therapy is based almost exclusively on the physician’s experience [3]. Researches has been focused mainly on the so called issue of ’closing the loop’ [4]. The idea is to create a system that can simulate the pancreas by automatic adjustment of insulin injections or, in some cases, by delivering glucagon to achieve the appropriate glucose level. The problem often requires building a decision-support system based on the prediction of the level of blood glucose.

In this paper, we propose a decision support tool for setting basal insulin therapy at the onset of type 1 diabetes. The proposed method is based on the concept of sequential patterns [5]. After determining the sequential patterns that underlie a given sequence of terms, it is possible to predict the plausible continuation of the sequence. From the medical perspective, sequential patterns can be treated as medical guidelines [3,6,7]. The advantage of using medical guidelines based on sequential patterns is that they are easy for physicians to interpret [8].

The concept of sequential patterns has been used extensively in different application domains [9], especially in medicine [10-12]. Sequential patterns have been used to identify diagnoses that diverse hospitals have in common [13] or to determine the subsets of examinations that were done together and that were followed by patients [14]. Several algorithms to discover sequential patterns have been proposed [5,15]. The concept of mining diabetes data using sequential patterns first was proposed in [16]. The patterns that were discovered were used to construct a decision tree that expressed the possible flow of medical events. Recently, [17] proposed the use of sequential patterns for planning diabetic therapy. The template-based patterns discovered from historical data were used to recommend the insulin dosage on the basis of the known value of the level of blood glucose [17].

One of the limitations of the approach proposed in [17] is the low generalization capability of the patterns that were discovered over the population of all patients. For that reason, the patterns were mined and used separately for each individual patient. In this paper, we describe our effort to extend the application of sequential patterns for supporting diabetic therapy by increasing their generalization capabilities. It is necessary to mention that such generalization is quite difficult for pre-meal insulin dosage, because each patient starts a meal with different BGL, and the measurement of the energy value of the meal is highly imprecise. For that reason, we left that challenge for future researches and focused our investigation in this research on mining patterns concerning only long-lasting basal insulin.

The contributions of this paper are as follows:

- A new approach for the qualitative evaluation of nocturnal glycaemia is introduced.
- The concept of differential sequential patterns and an algorithm for mining them are proposed.
- A decision support tool is developed based on differential sequential patterns. The decision tool’s output serves as a medical guideline for physicians when, the proper course of therapy must be determined quickly, e.g., for patients admitted to the hospital - new-onset diabetes.

The remainder of the paper is organized in the following way. We start with the Medical background section where the therapy of diabetes is presented. The basics of sequential patterns are given in Methods section, where later on the contribution of this paper is presented. First, the procedure of qualitative evaluation of nocturnal glycaemia is formalized. Second, the concept of differential sequences is introduced, and an algorithm for the generation of sequential patterns based on differential sequences is proposed. The validation of the proposed approach and the discussion of the results that were obtained are described in Results of experiments section.

## Medical background

The main goal of effective diabetes treatment is to maintain the state of near normoglyceamia. It is said that the normal glucose level should range between 70-140 mg/dl throughout the day, although the recommended range before a meal is 70-100 mg/dl [3]. Therefore repeated measurements of blood glucose during the day are performed [6]. Typically, the onset of type 1 diabetes among children is rapid, and such patients should be hospitalized. The initial daily insulin dosage is prescribed based on several basic data (e.g. the patient’s weight, gender and age), some test results (initial glicaemia level, acid-base balance, CRP, C-peptyd and HbA1c) and the proposed diet (patient’s energy demand). Currently, there is no strict medical procedure that allows immediately setting the proper insulin dosage based on the data listed above. Thus, in the following days, the daily glucose profile is taken, and the insulin dosage is adaptively modified. The daily glucose profile consists of glicaemia measurements every 2-3 hours during the night, fasting, before each meal, and 2 hours after each meal. The patient is released from the hospital when treatment is established, so that during the day, the blood glucose level is close to normal.

The overall daily insulin dosage is divided with 30% of the dosage administered as the basal dosage and 70% administered as the pre-meal insulin dosage. (Every patient with type 1 diabetes mellitus should take two types of insulin.) For the basal insulin the long-lasting (e.g., glargina insulin) is used. It is a biosynthetic recombinant analog insulin, that provides 24 hours of continuous activity, becomes fully acting as soon as 2 hours after administration, and is peakless. The basal insulin is usually injected in the evening (one injection per day), and it ensures the correct level of glucose during the night and before meals. In the following days, the results of current measurements of glucose are used to determine the dosage. The dose for the next day can be reduced when hypoglycemia occurred in the previous day, or it can be increased id hyperglycemia is observed in the glucose profile. In the case of increasing the glucose level (more than 200 mg/dl) during the night, the short acting insulin is administered immediately as the so-called ’basal correction’.

The second part of the therapy involves administering pre-meal insulin which, is short acting. This insulin is served just before each meal - usually 4-5 times a day.

Below, we provide example data concerning dosages of basal insulin and measurements of the levels of glucose in the blood. These data were collected during two exemplary days of a patient’s stay in the pediatric, endocrinology, and diabetology clinical hospital in Katowice, Poland. The data (Table 1) consist of the basal insulin dosage administered at 10 P.M. and four measurements of blood glucose level during the night (at 0:00, 3:00, 5:00, and 7:00 A.M.).

**Table 1:** **Exemplary clinical data**

**Patient**	**Date**	**Time[h]**	**Value**	**Description**
425	2008-11-26	22	10	Basal insulin
425	2008-11-27	0	109	Glycaemia
425	2008-11-27	3	62	Glycaemia
425	2008-11-27	5	60	Glycaemia
425	2008-11-27	7	71	Glycaemia
425	2008-11-27	22	9	Basal insulin
425	2008-11-28	0	114	Glycaemia
425	2008-11-28	3	112	Glycaemia
425	2008-11-28	5	105	Glycaemia
425	2008-11-28	7	99	Glycaemia
425	2008-11-29	22	8	Basal insulin

The nocturnal blood glucose level was interpreted in accordance with accepted medical standards [3] in the following way: when BGL is less than 70 mg/dl, it is treated as below normal; the normal level is between 70 and 140 mg/dl; the higher level (called mild-hyperglycemia) is recognized in the range of 140-200 mg/dl; and hyperglycemia is observed above 200 mg/dl. Thus, the corresponding symbolic values for the discretized glycaemia attribute are presented in Table 2.

**Table 2:** **Glycaemia discretisation**

**Blood glucose level [mg/dl]**	**Interpretation**	**Discrete value**
<70	Hypoglycemia	1
[70, 140)	Normoglycemia	2
[140, 200 ]	Mild-hyperglycemia	3
>200	Hyperglycemia	4

When planning therapy, the physician considers the patient’s clinical status and some test results [3], but the most important factor when the insulin dosage is initially specified is the patient’s weight. We used this indication such that the given basal insulin dosage is recalculated into units per 10 kg of the patient’s weight. For example, when considering the first day of therapy for patient 425, the basal insulin dosage is 10 units, and the patient’s weight is 32 kg. Thus, in our research the considered/normalized basal insulin dosage was rounded to 3 units per 10 kg.

## Methods

In this section, step-by-step we describe the way the decision support tool for therapy setting was created. We start with preliminaries about the notion of sequential patterns.

### The basics of sequential patterns

Let *E*={*e*_1_,*e*_2_,…,*e*_*n*_} denote the set of items (also called elements or events). Any subset of items $a_{i} \subseteq E$ is called an itemset. The sequence *a*=〈*a*_1_,*a*_2_,…,*a*_*k*_〉 is defined as an ordered list of itemsets *a*_*i*_. The term k-sequence denotes any sequence of cardinality *k*=*card*(*a*), where *k* is the number of itemsets it contains. The particular item *e*_*j*_∈*E* may recur in the k-sequence by being included in several itemsets *a*_*i*_.

The sequence *a*=〈*a*_1_,*a*_2_,…,*a*_*n*_〉 is contained [5] in the other sequence *b*=〈*b*_1_,*b*_2_,…,*b*_*m*_〉, i.e. $a \subseteq b$ if there exist integer numbers *i*_1_,*i*_2_,…,*i*_*n*_ such that $a_{1} \subseteq b_{i_{1}}, a_{2} \subseteq b_{i_{2}},\ldots,a_{n} \subseteq b_{i_{n}}$ [5]. The sequence *a* is called a subsequence of *b*, and the sequence *b* is called a supersequence of *a*.

Suppose the collection of sequences $S = \left \{ s^{1}, s^{2}, \ldots, s^{m} \right \}$ is available, where $s^{j} = \left \langle {s^{j}_{1}}, {s^{j}_{2}},\ldots, {s^{j}_{n}} \right \rangle $ denotes the *j*^*th*^ sequence consisting of ${s^{j}_{i}}$ itemsets. The support (1) of sequence *a* is a fraction of sequences in *S* that contain *a*.

(1) $$ sup(a) = \frac{card\left(s^{j} \in S |a \subseteq s^{j}\right)}{card(S)}  $$

The objective is to determine such subsequences $a \subseteq s^{j}$ that are frequently contained in any sequences from *S*, i.e., with the support *sup*(*a*)≥*sup*_*min*_, where *sup*_*min*_ is a threshold given by experts. Such sequences are in fact a kind of patterns in data, therefore they are called frequent patterns.

Several algorithms for mining frequent patterns have been proposed, the first and best known among them being AprioriAll and AprioriSome [5]. The goal of the other algorithms was to decrease the computational cost of mining [18-20]. An overview of sequential patterns mining algorithms is available in [21].

### Qualitative estimation of nocturnal glycaemia

In the case of the hospital for diabetic children that was considered, the measurements of BGL are made at 0:00, 3:00, 5:00 and 7:00 A.M. After numerous experiments, it became clear that the application of the arithmetic mean of four numerical measurements [17] was not fully correct and led to decreased support for the patterns that were discovered from the sequences stored as historical data. For that reason, we proposed a new approach.

In fact, qualitative evaluation of nocturnal glycaemia is usually done by doctors to estimate the level of glycamia during the night, and, further, it is used to determine the basal insulin dosage that is administered on the same day at 22:00 P.M. Formally, let us denote the sequence of BGL measurements at 0:00, 3:00, 5:00 and 7:00 as *bgl*=〈*bgl*_0_,*bgl*_3_,*bgl*_5_,*bgl*_7_〉, where *bgl*_*k*_ is a discrete value of the blood glucose level measured at time *k*∈{0:00, 3:00, 5:00, 7:00 }. Using that sequence, it is proposed that the level of nocturnal glycaemia be estimated by the following calculation:

$$  \textit{d} = \left\{ \begin{array}{ll} normoglycemia & \Leftrightarrow \forall_{k}(bgl_{k}=normoglycemia) \\ hypoglycemia & \Leftrightarrow \exists_{k}(bgl_{k}= hypoglycemia) \\ hyperglycemia & \Leftrightarrow \exists_{k}(bgl_{k}= hyperglycemia) \wedge \forall_{k}(bgl_{k} \neq hypoglycemia) \\ mild-hyperglycemia & otherwise\\ \end{array} \right.  $$

Table 3 illustrates the exemplary data given previously in Table 1 after the qualitative evaluation of nocturnal glycaemia. The qualitative evaluation is made in the column ‘Evaluation’, without any changes in the numerical values of the insulin dosage.

**Table 3:** **Exemplary clinical data after the qualitative evaluation of nocturnal glycaemia**

**Patient**	**Date**	**Time**	**Evaluation**	**Description**
425	2008-11-26	22	3	Basal insulin
425	2008-11-27	Night	Hypoglycemia	Glycaemia
425	2008-11-27	22	3	Basal insulin
425	2008-11-28	Night	Normoglycemia	Glycaemia
425	2008-11-28	22	3	Basal insulin

### Differential sequential patterns

Let us define an ordered pair *s*_*i*_=〈*z*_*i*_,*c*_*i*_〉, where *z*_*i*_ denotes the value of basal insulin dosage, and *c*_*i*_ is the value of nocturnal BGL. In this way every sequence *s*^*j*^=〈*s*_1_,*s*_2_,…,*s*_*n*_〉 is related to the basal-insulin therapy of the *j*^*th*^ patient. Every itemset within such sequence is, in fact, an ordered pair of events related to a single day of therapy. The goal of the therapy is to discover frequent patterns $p \subset s^{j}, j \in [1,n]$ that with high probability will recur for a newly-admitted patient, i.e., $p \subset s^{n+1}$.

As stated in Medical background, the basal-insuline therapy is often considered by physicians in terms of deviations of glycaemia and corresponding insulin dosages. To reflect this procedure, let us assume that the insulin dosage and the glycaemia level are denoted as *a* and *b*, respectively, at the day of patient’s admission; in this way, *s*_1_=〈*a*,*b*〉, where *a* and *b* are constants. The following insulin dosage (the next day) can be raised (denoted as ’+’), reduced (denoted as ’-’), or it can be kept at same level (denoted as ’0’). A similar operation is performed with the glycaemia level. For *i*≥2 we have:

$$ d_{i} = \left\{ \begin{array}{ll} - & \text{, for:}~~ z_{i}<z_{i-1}\\ 0 & \text{, for:}~~ z_{i}=z_{i-1} \\ + & \text{, for:}~~ z_{i}>z_{i-1} \\ \end{array} \right.  $$

$$ g_{i} = \left\{ \begin{array}{ll} - & \text{, for:}~~ c_{i} < c_{i-1} \\ 0 & \text{, for:}~~ c_{i} = c_{i-1} \\ + & \text{, for:}~~ c_{i} > c_{i-1} \\ \end{array} \right.  $$

To represent the therapy of a patient, we redefine a differential sequence as *S*=〈*s*_1_,*s*_2_,…,*s*_*n*_〉, where *s*_*i*_=〈*d*_*i*_,*g*_*i*_〉. For example, the sequence *S*=〈〈3,3〉,〈3,2〉,〈3,1〉,〈2,2〉〉 is converted to the differential sequence *D*=〈〈*a*,*b*〉,〈0,−〉,〈0,−〉,〈−,+〉〉 or shorter when omitting brackets *D*=〈*a*,*b*,*d*0,*g*−,*d*0,*g*−,*d*−,*g*+〉 - note that *d* stands for insulin dosage and *g* for glycaemia.

The standard support measure given by formula (1) is used for the evaluation of DSP.

### Discovering DSP from historical data

In this section an algorithm for mining DSP is proposed. The goal of the mining algorithm is to determine the set of differential patterns with the given support level. The input data for the algorithm are the set of differential sequences *S* prepared by pre-processing the clinical data gathered and interpreted according to description given in Qualitative estimation of nocturnal glycaemia.

First, the initial pattern candidate *a*_*k*_ is initiated with the itemset <*d*_0_,*g*_0_> from the first sequence from the set *S*. The support of the candidate pattern for each sequence in the set *S* is evaluated. Then, the pattern is enhanced with the following itemset <*d*_*j*_,*g*_*j*_> of the considered sequence, creating a new candidate pattern. The support of the new candidate pattern is evaluated, and the algorithm repeats. The loop is ended if the support of the candidate pattern is less than the given level *psup*_*min*_ or if there are no more itemsets in the sequence. The patterns with the support above *psup*_*min*_ and having the required length (here ≥6) are stored. The steps described above are repeated for each unique sequence from *S*.



The algorithm presented above (Algorithm ??) is based on the AprioriAll [5] sequential patterns discovery algorithm. There are many different variations of this algorithm implementation for different purposes with the task to reduce the time and memory computation complexity [22-24]. The algorithm above has been implemented by authors with worst time complexity not greater than *O*(*n*·*k*^2^) where *n* is the number of sequences and *k* is the average length of the sequences.

### Decision support

The main application of the mined DSPs is to support setting the therapy for a newly-admitted diabetic patient. The main idea of that support is to help the physician with the decision of changing the current treatment (increase or decrease the insulin dosage). That decision can be made with greater certainty by considering the knowledge gathered from similar cases presented in the form of differential patterns. In this way, the proposed supporting tool relies on the set of the mined differential patterns. The following steps lead the physician in determining the appropriate treatment for the patient:

1. The patient is admitted to the hospital cared for by the physician during a certain initial period (1-3 days)
2. The clinical data of the patient gathered during that initial period are converted into the form of differential sequence *a*^*j*^ (as described in this Section).
3. The computer program retrieves a subset of the patterns $S \subseteq P$ from the set of all available DSPs such that *a*^*j*^ is supported by each sequence from *S*, i.e., $S=\{s: a^{j} \subseteq s \wedge s \in P \}$. This set is called a set of treatment support patterns (TSP).
4. The selected patterns serve as the medical guidelines for the physician, supporting her or his decision regarding the following therapy.

After the patient’s hospitalization ends, all of the gathered data are added in the form of differential sequences to the set of all sequences and the algorithm for mining DSPs is run again.

## Results of experiments

The objective of the following experiments was to validate the theoretical approach proposed in this paper. We used the data of 102 children with onset of type 1 diabetes collected by the Department of Pediatrics, Endocrinology and Diabetology at the Silesian Medical University in Katowice, Poland. First, the data were processed, i.e., the discretization of BGL and insulin values was performed as described in Medical background. Afterwards the qualitative evaluation of nocturnal glycaemia was made.

The goal of the first experiment was to mine DSPs from the entire available dataset. By measuring the support, it was possible to check the generalization capabilities of the proposed DSP-based model over the set of all patients. The results are given in Table 4.

**Table 4:** **The set of DSPs mined from the entire set of data**

**DSP**	**Supp**
〈*a*,*b*,*d*0,*g*0,*d*0〉	0.39
〈*a*,*b*,*d*0,*g*−,*d*−〉	0.24
〈*a*,*b*,*d*0,*g*+,*d*0〉	0.23
〈*a*,*b*,*d*0,*g*0,*d*0,*g*0〉	0.16
〈*a*,*b*,*d*0,*g*0,*d*0,*g*−〉	0.15
〈*a*,*b*,*d*0,*g*0,*d*0,*g*0,*d*0〉	0.13
〈*a*,*b*,*d*0,*g*0,*d*0,*g*−,*d*0〉	0.13
〈*a*,*b*,*d*0,*g*−,*d*0,*g*0〉	0.13
〈*a*,*b*,*d*0,*g*+,*d*0,*g*0〉	0.13
〈*a*,*b*,*d*0,*g*−,*d*−,*g*+〉	0.11
〈*a*,*b*,*d*0,*g*−,*d*0,*g*+〉	0.11

Table 4 shows that the support of individual DSPs reached quite high values. After adding the support of the first three patterns that refer to alternative versions of therapy for the initial three steps, the cumulative support (0.39 + 0.24 + 0.23) = 0.86 was evaluated as high. This means that applying only three of the most general DSPs, it is possible to propose the therapy for 86% of the patients.

The objective of the second experiment was the evaluation of the usefulness of the mined DSPs for newly-admitted patients, and 5-fold cross validation with random sampling was applied. The set of patients’ sequences was partitioned into five parts. In each trial, the selected part served as the testing set, and the rest of sequences constituted the learning set. Differential patterns were mined from the learning set, and, then, their support in the testing set was calculated. The results of the experiment are presented in Tables 5 and 6.

**Table 5:** **Differential patterns, the results of 5-folded cross validation**

**1** ^***st***^ ** trial**	**Supp**	**2** ^***nd***^ ** trial**	**Supp**
〈*a*,*b*,*d*0,*g*0,*d*0〉	0.4	〈*a*,*b*,*d*0,*g*0,*d*0〉	0.5
〈*a*,*b*,*d*0,*g*−,*d*−〉	0.3	〈*a*,*b*,*d*0,*g*+,*d*0〉	0.3
〈*a*,*b*,*d*0,*g*0,*d*0,*g*0,*d*0〉	0.2	〈*a*,*b*,*d*0,*g*+,*d*0,*g*0〉	0.25
〈*a*,*b*,*d*0,*g*0,*d*0,*g*−〉	0.2	〈*a*,*b*,*d*0,*g*−,*d*−〉	0.2
〈*a*,*b*,*d*0,*g*0,*d*0,*g*−,*d*0〉	0.2	〈*a*,*b*,*d*0,*g*0,*d*0,*g*−〉	0.15
〈*a*,*b*,*d*0,*g*0,*d*0,*g*0〉	0.2	〈*a*,*b*,*d*0,*g*−,*d*−,*g*0〉	0.15
〈*a*,*b*,*d*0,*g*+,*d*0〉	0.15		
〈*a*,*b*,*d*−,*g*−,*d*+〉	0.15

**Table 6:** **Differential sequential patterns mined from every learning trial**

**3** ^***rd***^ ** trial**	**Supp**	**4** ^***th***^ ** trial**	**Supp**	**5** ^***th***^ ** trial**	**Supp**
〈*a*,*b*,*d*0,*g*0,*d*0〉	0.35	〈*a*,*b*,*d*0,*g*0,*d*0〉	0.35	〈*a*,*b*,*d*0,*g*0,*d*0〉	0.4
〈*a*,*b*,*d*0,*g*+,*d*0〉	0.25	〈*a*,*b*,*d*0,*g*+,*d*0〉	0.15	〈*a*,*b*,*d*0,*g*+,*d*0〉	0.3
〈*a*,*b*,*d*0,*g*0,*d*0,*g*−〉	0.15	〈*a*,*b*,*d*0,*g*−,*d*−,*g*+,*d*0〉	0.15	〈*a*,*b*,*d*0,*g*+,*d*0,*g*0〉	0.2
〈*a*,*b*,*d*0,*g*+,*d*0,*g*0〉	0.15	〈*a*,*b*,*d*0,*g*−,*d*−,*g*+〉	0.15	〈*a*,*b*,*d*0,*g*0,*d*0,*g*−,*d*0〉	0.15
〈*a*,*b*,*d*0,*g*+,*d*0,*g*0,*d*0〉	0.15	〈*a*,*b*,*d*0,*g*0,*d*0,*g*0〉	0.15	〈*a*,*b*,*d*0,*g*0,*d*0,*g*−〉	0.15

Similarly, as in previous experiment, the support that was obtained was evaluated as high. Taking into account the patterns with a minimal support level greater or equal to 0.2 and with the length of 2.5 days or more, the non-empty set of treatment support patterns was deduced in 89% of test examples in each trial. The mined patterns can be used effectively to support the therapy of newly-admitted patients.

We draw the following medical observations regarding the results that were obtained:

- The correction of the initially-administered insulin dosage is often not needed, e.g., the DSP =〈*d*0,*g*0,*d*0,*g*0,*d*0〉 represents that fact.
- The physician is not changing the dose hastily just after one day of observation, e.g., DSP =〈*g*+,*d*0〉, 〈*g*−,*d*0〉 is used, where g+,g- corresponds to the glycaemia level.
- The physician is trying to reduce the insulin dosage and maintain it at that level even if the body’s response is not clear, e.g, that is represented by DSP =〈*d*0,*g*−,*d*−〉.
- Considering the whole set of DSPs, it was apparent that the last dosage is usually the same as the previous dosage, meaning that the patient is leaving the hospital with the treatment settled and verified with the previous days.
- Another interesting observation is that the body’s response can change even when the insulin dosage is not changed, see, e.g. DSP =〈*d*0,*g*−,*d*0,*g*+〉.

## Conclusions

In this paper, the treatment of onset type 1 diabetes patients was analyzed. It was shown that the proposed differential sequential patterns for setting the basal insulin dosage can serve as guidelines for physicians during the day-after-day therapy. Also, by having these patterns, the physician has the opportunity to foresee the possible consequences of the prescribed therapy. Differential sequences can help the physician to decide the proper therapy faster. The usability of the proposed differential sequential patterns and the proposed mining algorithm was verified experimentally using real medical data. The results that were obtained provide evidence of the usefulness of the proposed approach.
